# Long-acting reversible contraception utilization and associated factors among women in extended postpartum period in southern Ethiopia

**DOI:** 10.1186/s13690-021-00683-4

**Published:** 2021-09-06

**Authors:** Yibeltal Mesfin, Abraham Wallelign

**Affiliations:** 1 Department of Midwifery, College of medicine and health sciences, Wolkite University, Wolkite, Ethiopia; 2 Department of Midwifery, College of medicine and health sciences, Debre Tabor University, Debre Tabor, Ethiopia

**Keywords:** Extended postpartum period, Long-acting reversible contraception, Postpartum women

## Abstract

**Background:**

Postpartum long-acting reversible contraceptive is important to prevent unintended and closed spaced pregnancy following the first 12 months of childbirth. Few data were available on postpartum long-acting reversible contraceptive use in Ethiopia. So, this study aimed to assess the long-acting reversible contraception use and associated factors among women who gave birth in the last 12 months.

**Methods:**

A community-based cross-sectional study was conducted from October 1st to November 2019. Systematic random sampling was applied to recruit a total of 416 study participants. Data were collected using a structured questionnaire. Data were entered using Epidata 4.6 and exported to SPSS version 25 for analysis. *P*-value < 0.05 with 95% confidence interval (CI) used to declare statistical significance.

**Result:**

In this study, long acting contraceptive utilization among women in the extended postpartum period was 22.6%. Maternal age ≤ 24 years (AOR = 3.7, 95% CI: 1.5, 8.9), being married (AOR =3.5 95% CI: (1.17–10.28)), menses resumption (AOR = 4.9 95% CI: (2.92, 8.20)), sexual intercourse resumption (AOR = 7.1 95% CI: (4.03, 12.56)) and received postpartum family planning counseling (AOR = 3.2 95% (1.95, 5.28)) were the factors associated with Long-acting reversible contraception use.

**Conclusion:**

This finding showed postpartum women’s long-acting reversible contraceptive use during the extended postpartum period was low. The factors significantly associated with extended postpartum modern contraceptive use were women’s age, being married, menses resumption, sexual intercourse resumption, and got postnatal family planning counseling. Strengthening Antenatal and postnatal counseling of postpartum family planning would improve long-acting reversible contraception use.

## Background

The long-acting reversible contraceptive (LARC) is a highly effective modern contraceptive method that includes an intrauterine contraceptive device (IUCD) and implants, which prevent unintended and closely spaced pregnancy [1]. Extended postpartum period LARC use refers to the initiation of IUCD or implants during the first 12 months of childbirth.

Globally, about 80 million unintended pregnancies occur during the postpartum period due to lower use of family planning [2]. In sub-Saharan African countries, 40% of unmet needs for family planning occur during the postpartum period [3]. The postpartum women LARC use has universally recognized several health benefits. LARC use reduces maternal and neonatal mortality and morbidity by preventing unintended pregnancy and unsafe abortion [4]. An individual couple delaying their next child at least 2 years and above can avert 30% of maternal and 10% of child mortality [5]. Published reports showed that a birth-to-pregnancy interval of above 2 years reduced prematurity, low birth weight, as well as small for gestational age [6]. In addition to health, FP has economic and social benefits for women, families, and the population at large. Family planning slowing the population growth, which aids the women to possess good income potential, dedicates enough for each child, resulting in reductions in poverty [7, 8].

Currently, family planning (FP) services incorporate into maternal and child health care services at all levels of the health care delivery system in Ethiopia. The government, local, and international partners on the FP program conducting maximal efforts to reach all over the country. The health extension workers intensively deliver the FP service to rural communities at health posts. [9]. Despite the above effort done, the Ethiopian demographic health survey (EDHS) 2016 reported only 7.1% of women utilized LARC, which was far from the global FP target 2030 [10]. A cross-sectional study in Hossana (36.5%) and Durame town (36.7%) of women were using long-acting reversible contraceptives during the extended postpartum period, which was higher than the national magnitude [11, 12].

Many previous studies identified factors influencing the poor uptake of the long-acting reversible modern postpartum contraceptive use in Ethiopia. Socio-demographic factors like age, level of education, marital status, and religion significantly affect LARC use [11, 12]. Antenatal and postnatal Family Planning counseling, menses return, resumption of sexual intercourse, and other maternal and reproductive health-related factors influence LARC utilization [13, 14].

Even though many studies were conducted to assess modern contraceptive use, there is a scarcity of evidence about factors affecting long-acting reversible contraceptive use during the extended postpartum period at the community level in southern Ethiopia. Therefore, this study aimed to assess the use of long-acting reversible contraceptives and associated factors among women who gave birth in the last 12 months in Arba Minch town, Southern Ethiopia.

## Methods

We conducted a community-based cross-sectional study design from October 1st to November 30th, 2019 in Arba Minch town. Arba Minch is the capital of the Gamo zone, southern nation nationalities peoples region, Ethiopia. Arba Minch Zuria woredas in the north, west, and south and Nech-Sar National park in the east and some part of the northeast are the border of the town. The total area of the town is 4011hactare structured with 11 kebeles. Arba Minch town has a population of 112,724, of which 56,137 (49.8%) are males and 56,587 (50.2%) are females. There is one General Hospital, three public health centers, and 11 health posts.

The source population of this study was all mothers who gave birth for the last 12 months before the study. The study population included sampled mothers who gave birth in the last 12 months before this study. The single population proportion formula was used to calculate the sample size. The assumption of 95% confidence level, 5% margin of error and prevalence of Long-acting reversible contraception use which is 36.7% (Durme town [15]) were considered. After adding a 20% non-response rate, the final computed sample size was 429.

We identified all households who have a mother with a history of birth in the previous year using the family folder of the Health extension. The proportional number of study participants was allocated for each kebele. A lottery method was used to select the first postpartum woman. Then, the next postpartum woman was selected through a systematic random sampling of every Kth (3rd) interval household. In a case when the study participants could not interview, we attempted two times to interview the respondent, then we asked the next study participant. We used the lottery method when over one mother who gave birth in the last 12 months was found in the same house.

### Data collection instrument and procedure

The data was collected by using a pre-tested structured questionnaire adapted from different literature, which was developed for similar purposes by different authors. The data collection tool (questionnaire) was prepared first in English and then translated to Amharic, which was translated back to English to ensure consistency. We used the Amharic version of data collection. The questionnaire comprises socioeconomic and demographic characteristics, reproductive history and maternal health care, knowledge, and current practice of long-acting reversible contraceptives during the extended postpartum period.

Two diploma midwives and two clinical nurses collected data. One BSc midwife supervisor the interview. We trained the interviewers for one day before the actual data collection. Lecturing, mock interviews, and actual field practice were used to train the data collectors. The interview was done under close supervision. Interviewer-administered questionnaires via face-to-face interviews were used for data collection.

### Data processing and analysis

All filed questioners were cheeked for completeness, consistency, and accuracy. The data was entered into Epi-Data version 4.6 and exported to the statical package of social science (SPSS) version 25 for further analysis.

Descriptive analyses (frequency, mean, percentage) were carried out for the variables. A Binary logistic regression was used to determine the relationship between the outcome and each independent variable. Those variables having a *P*-value of 0.25 and less in the bivariate analysis were added in the multivariable logistic regression analysis. Hosmer-Lemeshow goodness of fit test was used to check the fitness of the model, which did not have a statically significant *p*-value of 0.183. The adjusted odds ratio along with 95% of confidence interval was an estimate to identify factors associated with the outcome variable. The level of statistical significance was declared at a *p*-value less than 0.05. The results are finally presented in tables, graphs, and text.

## Result

### Socio-demographic characteristics of the respondent

A total of 416 postpartum women participated with a response rate of 96.96%. The age of women ranges from 17 to 45, with a mean age of 28.2 (SD ±4. 951) years. Three hundred ninety-two women (94.2%) were married (Table 1).

**Table 1:** Frequency distribution of postpartum women by their background characteristics in Arba Minch town, 2019(*N* = 416)

Variable	Number(n)	Percent (%)
**Age**		
≤ 24	92	22.2
25–34	276	66.3
≥ 35	48	11.5
**Marital status:**		
Married	392	94.2
Other*	24	5.8
**Women education**		
No formal education	67	16.1
Primary	122	29.3
Secondary	110	26.4
Diploma and above	117	28.2
**Partner education(*N*** **= 392)**		
No formal education	26	6.6
Primary	77	19.7
Secondary	133	33.9
Diploma and above	156	39.8
**Ethnicity**		
Gamo	229	55.1
Gofa	37	8.9
Wolayita	35	8.4
Amhara	68	16.3
Oromo	25	6.0
Others**	22	5.3
**Occupation**		
Housewife	105	25.2
Self-employed	110	26.4
Gov’t employed	103	24.8
Merchant	54	13.0
Others**	44	10.6
**Religion**		
Protestant	193	46.4
Orthodox	191	45.9
Others***	32	7.7
**Monthly income**		
≤ 1000	85	20.4
1001–2000	81	19.5
2001–3000	99	23.8
> 3000	151	36.3

Other *: Single, Divorced, Widwoid; Other**: Gurage, Tigre, Konso; Others** = Daily laborer, Unemployed; Others***: Muslim, Catholic

### Reproductive health-related characteristics of the study population

The median number of living children was 2.44 per woman. One hundred thirty-three (32.1%) had only one child. Seventy-three (17.5%) did not wish to have more children in the future. Three hundred fourteen (75.5%) of them had resumed sexual intercourse (Table 2).

**Table 2:** Distribution of postpartum women by reproductive health-related characteristics, Arba Minch town, 2019(*n* = 416)

Variable	Frequency (n)	Percent (%)
**Resumed Menses**		
Yes	234	56.2
No	182	43.8
**Resumed Sexual Intercourse**		
Yes	314	75.5
No	102	24.5
**Future Fertility Desire**		
Yes	343	82.5
No	73	17.5
**Postpartum period (wk.)**		
0–12	109	26.2
13–26	173	41.6
27–38	97	23.3
39–50	37	8.9

### Maternal health-related factors of the study population

The study indicated that women who had four or more ANC visits were 168 (40.4%). One-third (30.3%) of the respondents had a post-natal visit within 24 h. Two hundred forty-two (58.2%) women had family planning counseling during ANC and PNC (Table 3).

**Table 3:** Distribution of postpartum women by Maternal health-related factors, Arba Minch town, 2019(*n* = 416)

Variable	Frequency	Percent
**ANC Follow Up**		
No ANC follow up	30	7.2
< 4 Visits	218	52.4
4 and above visits	168	40.4
**PNC Follow Up:**		
Yes	126	30.3
No	290	69.7
**FP Counseling on ANC and PNC**		
Yes	242	58.2
No	174	41.8

### Male partner involvement related factors of the study population

Two hundred eighty-five postpartum women discuss with their partner. On the other hand, 68% of postpartum are supported by their partner to use the modern contraceptive.

### Knowledge and media exposure about contraceptive

Knowledge of at least one modern contraceptive is 98.1%. About the attitudes of the respondents towards the benefits of modern contraceptive utilization about half (50.7%) of them had positive attitudes. One hundred eighty-three (44.0%) postpartum women had got information from the media.

### Long-acting reversible contraceptive use in the extended postpartum period

**Fig. 1:**
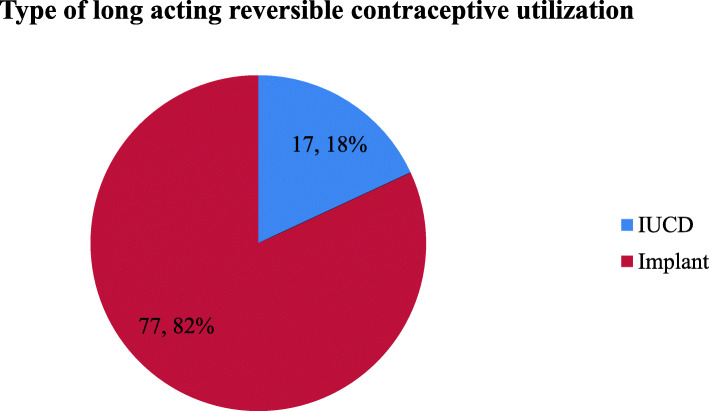
Magnitude of long-acting reversible contraceptive use by type of contraceptive among postpartum women in Arba Minch town, 2019(*N* = 94)

The prevalence of long-acting contraceptive utilization was found to be 94 (22.6%). Seventy-seven respondent used (82%) followed by IUCD 17(18%) (Fig. 1).

### Factors associated with postpartum long-acting reversible contraceptive utilization

In the multivariable logistic regression analysis, women’s age, being married, menses resumption, sexual intercourse resumption, and got family planning counseling showed a significant association with postpartum LARC use.

Maternal age ≤ 24 years (AOR = 3.7, 95% CI: 1.5, 8.9), being married (AOR = 3.5(1.17–10.28)) menses resumption (AOR = 4.9 95% CI: (2.92, 8.20)), sexual intercourse resumption (AOR = 7.1 95% CI: (4.03, 12.56)) times more likely to use postpartuim LARC compered to their counterpart.

The study also indicated that postpartum women who were counseled about postpartum family planning were 3.2 times more likely to use LARC than those who did not counsel [AOR = 3.2 95% (1.95, 5.28)] (Table 4).

**Table 4:** Factors associated with long-acting reversible contraceptive use during the extended postpartum period, Ariba Minch town, 2019 (*n* = 416)

Variable	COR (95%CI)	AOR (95%CI)	*P* value
Age of the women			
15–24	2.39 (1.17–4.87)	3.70 (1.52–8.91)	0.004
25–34	3.15 (1.68–5.90)	3.10 (1.44–6.68)	0.004
≥ 35	1.00		
Marital status			
Married	3.99 (1.66–9.55)	3.50 (1.17–10.28)	0.025
Others*	1.00		
Women Education:			
No formal education	1.00		
Primarily	1.42(.778–2.61)		
Secondary	1.63 (0.87–3.31)		
Diploma and above	2.29 (1.22–4.29)		
ANC Visits			
No visit	1.00		
< 4 visits	2.31 (1.06–5.02)		
4 and above	4.36 (1.94–9.79)		
Media Exposure			
Yes	2.63 (1.71–4.03)		
No	1.00		
PPFP Counseling			
Yes	4.00 (2.61–6.11)	3.20 (1.95–5.28)	0.0001
No	1.00		
Knowledge of PPFP			
Good	5.68 (1.13–8.51)		
Poor	1.00		
Menses Resumed:			
Yes	4.41 (2.87–6.68)	4.9 (2.92–8.20)	0.0001
No	1.00		
Sexual Intercourse resumed:			
Yes	6.73 (4.12–11.01)	7.10 (4.03–12.56)	0.0001
No	1.00		

Other*: single, divorced, widowed

## Discussion

This community-based research aimed to identify long-acting reversible contraceptives use and associated factors in Arba Minch town among women in the extended postpartum period. The overall prevalence of long-acting reversible contraceptive use was 22.5% during the extended postpartum period. This finding was higher than the national figure(7.1%) [16]. This may be justified by increased access to and inclusive sexual reproductive health services for the past 4 years.

The finding from Hosanna (36.5%) and Durame (36.7%) town were higher than this study [15, 17]. The reason for this difference might be due to the sample size, the residence of study participants, and access to information about family planning.

This study showed that postpartum women age less than 24 years were 3.7 times more likely to use LARC compared with women age 35 years and more. This finding is also supported by the study in Gondar Town, North West Ethiopia [18]. The possible reason may due to young women are more sexually active than older women.

This study also indicated that postpartum married women were three and half times more likely to use long-acting reversible contraceptives than others. This is consistent with the study done at Debre Tabor and Addis Ababa [19, 20]. This might be due to that married women were living with their husbands. They might start regular sexual intercourse earlier than the non-married couples that may force to use contraceptives to program the birth of the next child.

Family planning counseling during ANC and PNC was found to be associated with long-acting reversible contraceptive use during the extended postpartum period. Women who had received postpartum family planning counseling during ANC and PNC were 3.2 times more likely to use long-acting reversible contraceptives in the extended postpartum period than their counterparts. This result is agreed with Axum, north Ethiopia, and Malawi [12, 21]. This may be because women who have received family planning counseling during ANC and PNC might be highly motivated for LARC use.

Mothers whose menses resumed were almost five times more likely to utilize long-acting reversible contraceptives. This is inconsistent with the kinds of literature done in Addis Ababa and Gondar [18, 20]. This is because postpartum women may be aware of their fertility returning when menses return. This could be due to postpartum women believed that a return of fertility only occurs along with the return of menses.

The other stronger factor for the need for family planning is the resumption of sexual intercourse. Women who have resumed sexual intercourse were almost seven times more likely to use LARC compared to counterparts. This finding is supported by a study done by Hosanna and Axum [11, 12]. This might be because women who resumed sexual activity have a fear of getting pregnant. Consequently; they seek contraception than those who had not resumed sex.

## Conclusion and recommendations

The finding of this showed that the level of long-acting reversible contraceptive utilization in the extended postpartum period was low. Women’s age, being married, menses resumption, resumption of sexual intercourse, and family planning counseling during ANC and PNC were significant factors associated with the long-acting reversible contraceptive use. Therefore, health institutions should strengthen antenatal and postnatal counseling of postpartum family planning. Health workers also should provide advice and counseling for postpartum women that make them understand that fertility may precede the return of menses.

## Data Availability

Full data set and other materials relating to this study can be obtained from the corresponding author upon reasonable request.

## Abbreviations

AOR: Adjusted odds ratio
EDHS: Ethiopian demographic health survey
CI: Confidence interval
COR: Crude odds ratio
PPFP: Postpartum Family Planning
FP: Family Planning
HSDP: Health Sector Development Program
LARC: long-acting reversible contraceptive
WHO: World Health Organization

## References

[1] Programming strategies for postpartum family planning. WHO. 2013 [cited 2019]. Available from: https://www.who.int/reproductivehealth/publications/family_planning/ppfp_strategies/en/.

[2] Bellizzi S, Mannava P, Nagai M, Sobel H (2020). Reasons for discontinuation of contraception among women with a current unintended pregnancy in 36 low and middle-income countries. Contraception..

[3] Khan MN, Harris ML, Shifti DM, Laar AS, Loxton D (2019). Effects of unintended pregnancy on maternal healthcare services utilization in low-and lower-middle-income countries: systematic review and meta-analysis. Int J Public Health.

[4] Kinney MV, Kerber KJ, Black RE, Cohen B, Nkrumah F, Coovadia H, Nampala PM, Lawn JE, Axelson H, Bergh AM, Chopra M, Diab R, Friberg I, Odubanjo O, Walker N, Weissman E, Science in Action: Saving the lives of Africa's Mothers, Newborns, and Children working group (2010). Sub-Saharan Africa's mothers, newborns, and children: where and why do they die?. PLoS Med.

[5] Ganatra B, Faundes A (2016). Role of birth spacing, family planning services, safe abortion services and post-abortion care in reducing maternal mortality. Best Pract Res Clin Obstet Gynaecol.

[6] Bater J, Lauer JM, Ghosh S, Webb P, Agaba E, Bashaasha B, Turyashemererwa FM, Shrestha R, Duggan CP (2020). Predictors of low birth weight and preterm birth in rural Uganda: findings from a birth cohort study. PLoS One.

[7] Allison A, Basikoro EE (2017). Why world vision supports healthy timing and spacing of pregnancies to improve maternal and child health: a faith-based perspective. Christian J Global Health.

[8] Norton M, Chandra-Mouli V, Lane C (2017). Interventions for preventing unintended, rapid repeat pregnancy among adolescents: a review of the evidence and lessons from high-quality evaluations. Glob Health Sci Pract.

[9] Tigabu S, Demelew T, Seid A, Sime B, Manyazewal T (2016). Access to and utilization of quality family planning services: challenges and opportunities in meeting FP2020 targets. IJHSR..

[10] Csa I. Central statistical agency (CSA)[Ethiopia] and ICF. Ethiopia demographic and health survey. Addis Ababa, Ethiopia and Calverton, Maryland; 2016.

[11] Gejo NG, Anshebo AA, Dinsa LH (2019). Postpartum modern contraceptive use and associated factors in Hossana town. PLoS One.

[12] Abraha TH, Teferra AS, Gelagay AA. Postpartum modern contraceptive use in northern Ethiopia: prevalence and associated factors. Epidemiol Health. 2017;39. 10.4178/epih.e2017012 PMC5434225 28330336

[13] Ashebir W, Tadesse T. Associated factors of postpartum modern contraceptive use in Burie District, Amhara region, Ethiopia. J Pregnancy. 2020;2020. 10.1155/2020/6174504 PMC7115150 32257443

[14] Mengesha ZB, Worku AG, Feleke SA (2015). Contraceptive adoption in the extended postpartum period is low in Northwest Ethiopia. BMC Pregnancy Childbirth.

[15] Tamrie YE, Hanna EG, Argaw MD (2015). Determinants of long acting reversible contraception method use among mothers in extended postpartum period, Durame town, southern Ethiopia: a cross sectional community based survey. Health..

[16] Mansori K, Hanis SM, Shadmani FK. Postpartum modern contraceptive use in northern Ethiopia: prevalence and associated factors-methodological issues in this cross-sectional study. Epidemiol Health. 2017;39. 10.4178/epih.e2017019. 10.4178/epih.e2017019 PMC5543293 28774166

[17] Woldu BF, Ermolo TL, Lemu LG, Gejo NG (2020). Long-acting reversible contraception utilization and associated factors among women in extended postpartum period in Hossana town, southern Ethiopia: cross sectional study. Contracept Reprod Med.

[18] Abera Y, Mengesha ZB, Tessema GA (2015). Postpartum contraceptive use in Gondar town, Northwest Ethiopia: a community based cross-sectional study. BMC Womens Health.

[19] Taye EB, Mekonen DG, Debele TZ (2019). Prevalence of post partum modern family planning utilization and associated factors among postpartum mothers in Debre Tabor town, north West Ethiopia, 2018. BMC Res Notes.

[20] Gebremedhin AY, Kebede Y, Gelagay AA, Habitu YA (2018). Family planning use and its associated factors among women in the extended postpartum period in Addis Ababa, Ethiopia. Contracept Reprod Med.

[21] Bwazi C, Maluwa A, Chimwaza A, Pindani M (2014). Utilization of postpartum family planning services between six and twelve months of delivery at Ntchisi District hospital, Malawi. Health.

